# A novel dental infiltration resin based on isosorbide-derived dimethacrylate with high biocompatibility, hydrolysis resistance, and antibacterial effect

**DOI:** 10.3389/fbioe.2022.1049894

**Published:** 2022-11-10

**Authors:** Su Yang, Baiyan Sui, Yinan Cui, Xin Liu, Jiao Sun, Jun Wang

**Affiliations:** ^1^ Department of Pediatric Dentistry, Shanghai Ninth People’s Hospital, Shanghai Jiao Tong University School of Medicine, College of Stomatology, Shanghai Jiao Tong University, National Center for Stomatology, National Clinical Research Center for Oral Diseases, Shanghai Key Laboratory of Stomatology, Innovative Research Team of High-Level Local Universities in Shanghai, China; ^2^ Department of Dental Materials, Shanghai Biomaterials Research and Testing Center, Shanghai Ninth People’s Hospital, Shanghai Jiao Tong University School of Medicine, College of Stomatology, Shanghai Jiao Tong University, National Center for Stomatology, National Clinical Research Center for Oral Diseases, Shanghai Key Laboratory of Stomatology, Shanghai, China

**Keywords:** microinvasive treatment, dental caries, enamel demineralization, infiltration resin, isosorbide, biobased monomer

## Abstract

**Objectives:** The available infiltration resin has raised biosafety and treatment stability concerns because of the cytotoxicity of the main component, TEGDMA, and its susceptibility to hydrolysis in the oral environment. This study aimed to develop a TEGDMA-free infiltration resin to overcome these drawbacks.

**Methods:** Using the synthetic bioderived monomer bis(methacrylate) isosorbide (IBM) and the zwitterionic compound 2-methacryloyloxyethyl phosphorylcholine (MPC), a novel infiltrant IBMA was developed and preferentially selected. We investigated the performance of the IBMA resin regarding cytotoxicity, antibiofilm adhesion, and hydrolysis resistance and further verified its ability to restore the demineralized enamel and stability of the infiltrated area under artificial aging conditions.

**Results:** Compared with the commercial TEGDMA-based infiltration resin ICON, IBMA not only demonstrated similar enamel morphologic and esthetic restorative effects in chalky lesions but also exhibited favorable cell viability, durable *Streptococcus mutans* UA159 biofilm-repellent performance, and higher enamel microhardness (204.0 ± 5.12 HV) of the infiltrated enamel. Specifically, because of the high crosslink density [(47.77 ± 5.76) ×10^3^ mol/mm^3^] and low water sorption [12.79 ± 2.56 µg/mm^3^] of the polymer network, the IBMA resin was more resistant to hydrolysis than ICON, which prevents the disruption of the infiltrant’s micropore-blocking effect after aging. Enamel lesions treated with IBMA demonstrated good color stability after the tea-staining challenge, which was significantly better than that in the ICON group.

**Conclusion:** Based on these findings, the IBMA resin exhibits favorable cell viability, hydrolysis resistance, and biofilm-repellent properties, which alleviates the defects of traditional TEGDMA systems. Therefore, it is a better alternative for microinvasive treatment involving early caries and enamel whitish discoloration.

## 1 Introduction

In modern dentistry, minimally invasive treatment is gaining popularity because it preserves dental tissue to the greatest extent and effectively reduces patients’ anxiety. As a microinvasive technique, the existing infiltration resin can inhibit the progression of early caries and improve the esthetics of whitish discoloration (Eckstein et al., 2015; Borges et al., 2016; Peters et al., 2019; Olmo-González et al., 2020). However, its main component, TEGDMA, is easily released from the matrix and causes cytotoxic reactions (Geurtsen and Leyhausen, 2001; Bakopoulou et al., 2011), highlighting the need to pay specific attention to infiltration resin’s biosafety issues since it is commonly used to treat children and adolescents (Gölz et al., 2016). Moreover, the material is prone to degradation under oral conditions (Bourbia and Finer, 2018), which would undermine its caries prevention effect and result in failure of the treatment. Clinically, these problems can be attributed to TEGDMA’s characteristics. It is a long-chain monomer with excellent flexibility and contains triethylene glycol hydrophilic groups that are susceptible to water sorption and ester bond breakage (Chen et al., 2019; Pratap et al., 2019). Although some studies have attempted to improve the hydrolytic stability by adding hydrophobic monomers, such as BIS-EMA and UDMA (Araújo et al., 2013), these efforts cannot address the inherent drawbacks of TEGDMA-based resin materials. Therefore, it is necessary to introduce another monomer as the main component to develop a novel TEGDMA-free infiltration resin with excellent biocompatibility and hydrolysis resistance.

The low viscosity of the infiltration resin is critical for hindering early caries progression and improving the enamel color as the low-viscous resin can penetrate and fill the microporous area of the demineralized lesion *via* capillary force and form a resin-porous hydroxyapatite crystal complex (Paris et al., 2007a; Paris et al., 2007b). Eventually, the barrier structure prevents the entry of cariogenic factors and restores the translucent appearance of chalky enamel by increasing the local refractive index (Paris et al., 2013). Isosorbide is a non-toxic glycol derived from glucose and has been deemed “generally recognized as safe” (GRAS) by the U.S. Food and Drug Administration (Fenouillot et al., 2010). Some studies have synthesized a variety of resin monomers derived from isosorbide and used them to develop injectable bone cement materials with a high cell viability (Łukaszczyk et al., 2014). Herrera-González et al. also utilized bis(methacrylate) isosorbide (IBM) as the diluent in composite resins (Herrera-González et al., 2019). From the literature, we realized that the biobased IBM monomer has a low viscosity similar to that of TEGDMA. In addition, considering that IBM has a shorter molecular chain with the rigid dual five-membered ring structure, the IBM-based polymer’s crosslink degree would theoretically be denser than that of long-chain TEGDMA (Park et al., 2011; Gajewski et al., 2012), from which we speculated that it may exhibit better hydrolysis resistance than the TEGDMA system. Therefore, the low-viscosity bioderived photocurable monomer IBM was initially introduced as TEGDMA’s substitution to enhance the biosafety and antihydrolysis property of the infiltration resin.

The accumulation of oral plaque adversely affects the clinical performance of the infiltration resin in demineralized lesions over time since bacterial acid could cause enamel demineralization and the release of esterases accelerates the degradation of the resin matrix (Kielbassa et al., 2020). Several studies have modified the infiltration resin with chlorhexidine, nanosilver particles, and other release-type antibacterial ingredients (Inagaki et al., 2016; Angel Villegas et al., 2019; Kielbassa et al., 2020); however, the short-term burst effect would undermine the material’s stability and long-term antibacterial effect. In addition, contact-type antibacterial activities of quaternary ammonium salt monomers could be limited because of the interference of salivary protein adhesion (Yu et al., 2020). Considering that protein adsorption is the initial step of bacterial colonization, protein-repellent zwitterionic compounds may prevent the formation of plaque at the source. Among these, 2-methacryloyloxyethyl phosphorylcholine (MPC) is used in oral fluids, adhesives, and dental fillings (Zhang et al., 2015a; Zhang et al., 2015b) since ample free water around phosphorylcholine groups detaches protein effectively (Cheng et al., 2017). Moreover, its double bonds contribute to the formation of photocurable polymer networks, resulting in the long-lasting antibacterial activity (Zhang et al., 2018). In addition, MPC exhibits good biocompatibility since it is prepared by imitating the outer structure of the cell membrane, which is in line with our high-biosafety design philosophy of the new infiltration resin.

In brief, based on the rational assumption of polymer characteristics and the strategy of antibiofilm adhesion, this study innovatively utilized the biobased IBM monomer as the main component and a small amount of MPC to develop a novel infiltration resin with good biocompatibility, hydrolysis resistance, and antibacterial property, which is expected to provide a better minimally invasive treatment for patients with early caries and chalky discolored teeth, especially children and adolescents.

## 2 Materials and methods

### 2.1 Preparation and preference of IBM-based antibacterial infiltration resins

#### 2.1.1 Synthesis and characterization of the IBM monomer

The materials and solvents used in the synthesis were purchased from Sigma-Aldrich (St. Louis, United States). In a 250-ml three-necked flask, isosorbide (5.01 g, 0.034 mol), triethylamine (14.56 g, 0.144 mol), and dimethylolpropionic acid (DMAP) (0.42 g, 3.4 mmol) were dissolved using 30 ml anhydrous CH_2_Cl_2_ under magnetic stirring. The reaction flask was set in an ice water bath, and methacryloyl chloride (11.92 g, 0.114 mol) was added dropwise. Next, the temperature was naturally raised to room temperature, and the reaction was carried out overnight. Water was added to quench the reaction, the organic phase was separated, and the aqueous phase was extracted once using CH_2_Cl_2_. Combined organic phases were dried using anhydrous Na_2_SO_4_ and then spin-dried. By silica gel column chromatography, the crude product was purified [eluent: n-hexane/dichloromethane/methanol (v/v/v = 100/300/5)] to yield 5.14 g of the transparent liquid bis(methacrylate) isosorbide (IBM). The chemical reaction process of the synthesis is shown in Supplementary Figure S1.

Synthetic IBM was confirmed by ^1^H nuclear magnetic resonance (^1^H NMR). The NMR spectra were obtained with an NMR spectrometer (ECZ 400YH, JEOL), using deuterated chloroform (CDCl_3_) and tetramethylsilane (TMS) as the solvent and internal reference, respectively. The viscosity of IBM was determined using a rotary rheometer (MCR302, Anton Paar) equipped with the cone plate CP25. Rheological measurements were performed for shear rates ranging from 0.1 to 10 s^−1^ at a constant temperature of 37°C.

#### 2.1.2 Preparation of IBM-based infiltration resins

2-Methacryloyloxyethyl phosphorylcholine (MPC), camphor quinone (CQ), and dimethyl aminoethyl methacrylate (DMAEMA) as the coinitiators and butylated hydroxytoluene (BHT) as the inhibitor were purchased from Sigma-Aldrich (St. Louis, United States). Four experimental IBM-containing resins were fabricated, according to the mass fractions shown in Table 1. The mixture was magnetically stirred in the dark for 30 min. Prior to use, the resins were stored in the dark at 4°C in opaque recipients in order to prevent premature polymerization. The commercial infiltrant ICON (DMG, Germany) was used as a control group.

**TABLE 1:** Composition of IBM-based infiltration resins.

	IBM (%)	MPC (%)	CQ (%)	DMAEMA(%)	BHT (%)
IBM resin	98.4	0	0.5	1	0.1
IBM + 1% MPC resin	97.4	1	0.5	1	0.1
IBM + 2% MPC resin	96.4	2	0.5	1	0.1
IBM + 3% MPC resin	95.4	3	0.5	1	0.1

#### 2.1.3 Protein adsorption and antibacterial activity against the *S. mutans* biofilm of different resins

Cured samples of four IBM-based infiltration resins and the ICON resin were produced using disc-shaped metallic molds of 15 mm ranging 1 mm in diameter and thickness, respectively. The molds were filled up with each material, covered with polyester strips and a glass slide, and finally light-cured for 40 s using a dental curing unit (Elipar DeepCure-S, 3M ESPE). The amount of protein adsorbed on the disc was determined by using the micro-bicinchoninic acid (BCA) method (details are provided in the Supplementary Material).

*S. mutans* UA159 frozen in skim milk was cultured in the brain heart infusion (BHI, BD-Difco) medium containing 1% sucrose (S8270; Solarbio) at 37°C in a facultative anaerobic environment with 5% CO_2_. The bacterial suspension was then diluted with a sterile medium to a concentration of 10^6^ CFU/ml before use. Disk-shaped cured specimens of different resin groups (N = 10) were placed in sterile 12-well plates. In addition, 2 ml of the BHI medium and 20 ul of the diluted UA159 suspension were added to each well. After incubation for 24 h, the specimens were washed three times with phosphate-buffered saline (PBS) to remove the residual planktonic bacteria and the culture medium. The level of biomass accumulation on each specimen was determined through a crystal violet assay (details are provided in the Supplementary Material).

For scanning electron microscopy (SEM) analysis, four discs with the UA159 biofilm were immersed in 3% glutaraldehyde (Solarbio) overnight at 4°C. Next, the disks were washed twice with PBS and dehydrated *via* a graded ethanol series (50%, 70%, 80%, 90%, 95%, and 100%; 10 min immersion time per solution), followed by sputter coating with gold. Finally, the biofilms were examined by field-emission SEM (Mira3, Tescan) at a working voltage and magnification of 5 kV and 5 kx, respectively.

#### 2.1.4 Assessment of cell viability of different resins

Based on the ratio of 0.2 g/ml, the cured samples were immersed in DMEM (Gibco) containing 10% FBS and 1% PS for 24 h at 37°C to obtain the conditioned medium of each group. After 24 h of the culture being exposed to different conditioned mediums, the viability of L929 cells was assessed using a CCK-8 assay (Cell Counting Kit-8; Dojindo). A total of six replicates were tested for each group.

### 2.2 Hydrolysis-resistant performance of the IBMA resin under simulated aging

#### 2.2.1 Evaluation of the crosslink density and water sorption of different resins

Three cured specimens of each group (ICON, IBM, and IBMA resins) were prepared, as described previously (in Section 2.1.3), using the rectangular metallic mold (height: 1 mm, width: 5 mm, and length: 30 mm). In addition, they were analyzed using DMA 850 (TA Instruments) in the tensile mode with a frequency of 1 Hz, amplitude of 3 um, and a temperature range of 30–160°C at a speed of 3°C/min. The tangent curve, storage modulus curve (E′), and loss modulus curve (E″) were obtained, as shown in Supplementary Figure S3. The direct crosslink density equals E/(6RT) (Lin et al., 2012), and E refers to the storage modulus at Tg+40°C.

The cured samples of each group (N = 6) were prepared, as described previously, using the cylindrical mold (height: 6 mm and diameter: 4 mm). Using a Vickers indenter (HXD-1000TMC/LCD, Taming), microhardness measurements were taken on four quadrants of the surface under a load of 50 g for 10 s. The initial Vickers microhardness number (VHN_1_) was the average of four indentations. Afterward, the specimens were stored in absolute ethanol for 24 h at room temperature, and microhardness was again detected as VHN_2_. The softening ratio was determined using the equation (VHN_1_-VHN_2_)/VHN_1_ × 100%, which is considered an indirect estimation of the crosslink density. Water sorption tests were based on ISO 4049: 2009 specifications (details are provided in the Supplementary Material).

#### 2.2.2 ATR–Fourier infrared spectroscopy of different resins before and after thermocycling

Cylindrical samples of each group (N = 3) were prepared, as detailed in Section 2.2.1. Artificial aging was carried out using a thermocycling machine (TC-501F, Weier). The samples were immersed in cold water at 5°C for 30 s, followed by immersion in hot water at 55°C for 30 s. This cycle was repeated 5,000 times. Chemical properties before and after thermocycling were examined by Attenuated Total Reflectance-Fourier Transform Infrared spectroscopy (ATR-FTIR) spectroscopy (Nicolet iS10; Thermo Fisher Scientific). Infrared spectra were recorded under the following conditions: a resolution of 4 cm^−1^ with 32 time scans, 400–4,000 cm^−1^ wave number range, and a 25 ± 1°C chamber temperature. -OH stretching vibrations at approximately 3,400 cm^−1^.

#### 2.2.3 Scanning electron microscopy observation of different resins before and after thermocycling

Six cured specimens of each group were obtained, as detailed in Section 2.2.2, half of which served as a baseline, while the other half was subjected to thermocycling aging. Afterward, the samples were dried and sprayed with gold. Surface morphology was observed by field emission SEM (Mira3, Tescan) at a working voltage and magnification of 5 kV and 10 kx, respectively.

### 2.3 Scanning electron microscopy analysis of biofilm-repellent performance of different resins under simulated aging

The cured specimens of ICON, IBM, and IBMA resins (N = 6) were obtained, as detailed in Section 2.1.3, half of which served as a baseline, while the other half was subjected to thermocycling aging. Next, all samples of each group were placed in sterile 12-well plates. UA159 biofilms were cultured on discs, as explained in Section 2.1.3 for 24 h. Then, PBS-rinsed discs of each group were fixed, dehydrated, and sprayed with gold. Finally, biofilm adhesion was assessed using a field emission SEM (Mira3, Tescan) at a working voltage and magnification of 5 kV and 5 kx, respectively.

### 2.4 Infiltration performance and recovery effect of the IBMA resin on the demineralized enamel

#### 2.4.1 Confocal laser scanning microscopy evaluation of infiltration depth on the demineralized enamel

Experimental details are presented in the Supplementary Material. Infiltrated enamel slices were observed by confocal laser scanning microscopy (CLSM) (SP8, Leica) using a 20* objective. Owing to the staining and bleaching procedure of the samples, only resin-infiltrated parts, where the TRITC was enclosed by the resin, showed red fluorescence. In confocal microscopic images, infiltration depths were measured at five predefined points distributed equally along the lesion, which were defined as the distance from the surface to the deepest point of red fluorescence.

#### 2.4.2 Resin infiltration on the demineralized enamel

Demineralized lesions were treated with 15% hydrochloric acid etching for 2 min, rinsed with air–water spray for 30 s, and air-dried for 10 s. Afterward, the etched surface was wetted with absolute ethanol for 30 s and dried for 10 s. Each infiltrant was applied to the demineralized zone for 5 min, and the excess material was removed using cotton rolls. The resin-infiltrated surface was then light-cured for 40 s, followed by 20 s of polishing with 4000-grit aluminum oxide abrasive paper.

#### 2.4.3 Surface morphology of the infiltrated enamel and Vickers microhardness assessments

Demineralized lesions infiltrated with different resins were obtained, as detailed in Section 2.4.2. In addition, untreated demineralized specimens and sound enamel blocks served as controls. The samples were dried and sprayed with gold, and surface morphology was observed by field emission SEM (Mira3, Tescan) at a working voltage and magnification of 5 kV and 20 kx, respectively. Vickers microhardness of different infiltrated lesions and untreated demineralized specimens (N = 10) was measured as 2.2.1 under a 50 g load for 15 s. Furthermore, Vickers microhardness of cured specimens (N = 6) from different resins was measured using a 50 g load for 10 s.

#### 2.4.4 Color evaluation

Specimens with the demineralized enamel were obtained and randomly divided into three groups (N = 20), namely, ICON, IBM, and IBMA resins. Before and after resin infiltration, the color evaluation of specimens was performed using a dental spectrophotometer (Easyshade V, VITA Zahnfabrick), following the CIE L*a*b* system (details are provided in the Supplementary Material). The recently recommended CIE L*a*b* color space-based whitening index (WI_D_) was calculated according to the formula (Pérez et al., 2016): WI_D_ = 0.511L * −2.324a * −1.100b*. ΔL, Δa, Δb, and whiteness variation ΔWI_D_ indicate differences in each value between baseline and post-operation. It was suggested that ΔWI_D_ should be interpreted based on the whiteness 50%:50% perceptibility (WPT = 0.72 WI_D_ units) and 50%:50% acceptability (WAT = 2.62 WI_D_ units) thresholds (Pérez et al., 2018). The images of each sample were captured using a digital camera (Canon EOS 700D) under standardized lighting conditions, distance, and exposure.

### 2.5 Surface morphology and color stability of IBMA-infiltrated areas after simulated aging

#### 2.5.1 Surface morphology of the infiltrated enamel before and after thermocycling

Demineralized lesions infiltrated with different resins were obtained, as explained in Section 2.4.2, half of which served as a baseline, while the other half was subjected to thermocycling. The microstructures of the resin-infiltrated enamel before and after aging challenges were observed using SEM. Finally, micrographs were collected at 5 kV voltage and 5 kx magnification.

#### 2.5.2 Color evaluation of the infiltrated enamel after the staining challenge

Demineralized lesions infiltrated with different resins and sound enamel blocks were subjected to thermocycling. They were then immersed in a solution of 2% black tea for 2 h/day for 7 days at 37°C. Then, 2% black tea was produced by the filtration of 2 g of tea (Qimen black tea) in 100 ml of boiling water for 5 min. The measurement of the color change before and after staining was carried out as detailed in Section 2.4.4, and the difference in whiteness index was recorded as ΔWI_D_, which was finally assessed through comparisons with 50%:50% WPT and WAT, as mentioned previously.

### 2.6 Statistical analysis

Statistical analysis was performed by SPSS 22.0 software for WINDOWS (IBM, United States). All data were tested for normal distribution and homogeneity of variance. The results of protein adsorption among groups were analyzed using Brown–Forsythe and Welch ANOVA tests. Other comparisons among different resins were conducted by one-way analysis of variance (ANOVA), followed by multiple comparisons test. For all statistical analyses, the significance level was set at 5%. The values are presented as the mean ± SD.

## 3 Results

### 3.1 Preparation and preference of IBM-based antibacterial infiltration resins

**FIGURE 1:**
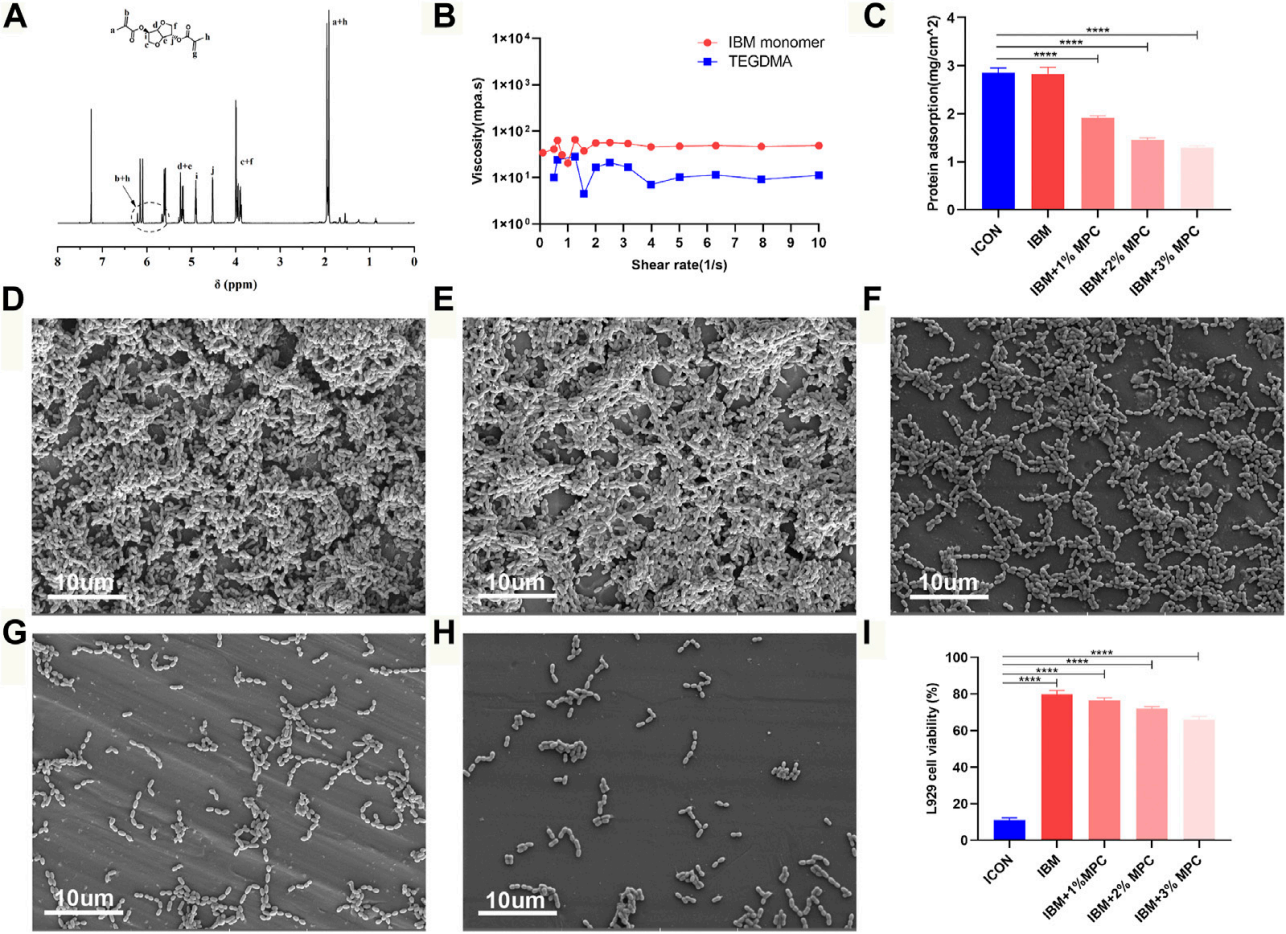
**(A)** NMR spectra of the IBM monomer. **(B)** Viscosity of the IBM monomer and TEGDMA. **(C)** Protein adsorption on IBM-based infiltration resins and the ICON resin. SEM image of the UA159 biofilm on the surface of the ICON resin **(D)**, IBM resin **(E)**, IBM + 1%MPC resin **(F)**, IBM + 2%MPC resin **(G)**, and IBM + 3%MPC resin **(H)**. **(I)** Cell viability of IBM-based infiltration resins and the ICON resin. Error bars represent SD.****p < 0.0001.

The IBM monomer was synthesized from isosorbide and methacryloyl chloride (Supplementary Figure S1), and its structure was confirmed by NMR analysis (Figure 1A). The viscosity value of the IBM monomer was 49 mPa s at a shear rate of 10 s^−1^, which was slightly higher than that of TEGDMA (Figure 1B). Protein adsorption by the IBM-based resin containing 1%, 2%, and 3% MPC was 1.91 ± 0.04 mg/cm^2^, 1.45 ± ±0.05 mg/cm^2^, and 1.29 ± 0.04 mg/cm^2^, respectively, which was lower than that in ICON (2.85 ± 0.10 mg/cm^2^), and the difference was statistically significant (*p* < 0.0001) (Figure 1C). UA159 biofilm adhesion on the surface of each group is shown in Figures 1D–H. When the MPC content was increased, a significant decrease in bacterial adhesion on the surface was observed. Cell viability of each IBM-based infiltration resin was higher than that of ICON, and the difference was statistically significant (*p* < 0.0001) (Figure 1I). The IBM-based resin containing 0%, 1%, and 2% MPC showed more than 70% cell viability, while that of the ICON resin was only 11.22 ± 1.08%.

### 3.2 Hydrolysis-resistant performance of the IBMA resin under simulated aging

**FIGURE 2:**
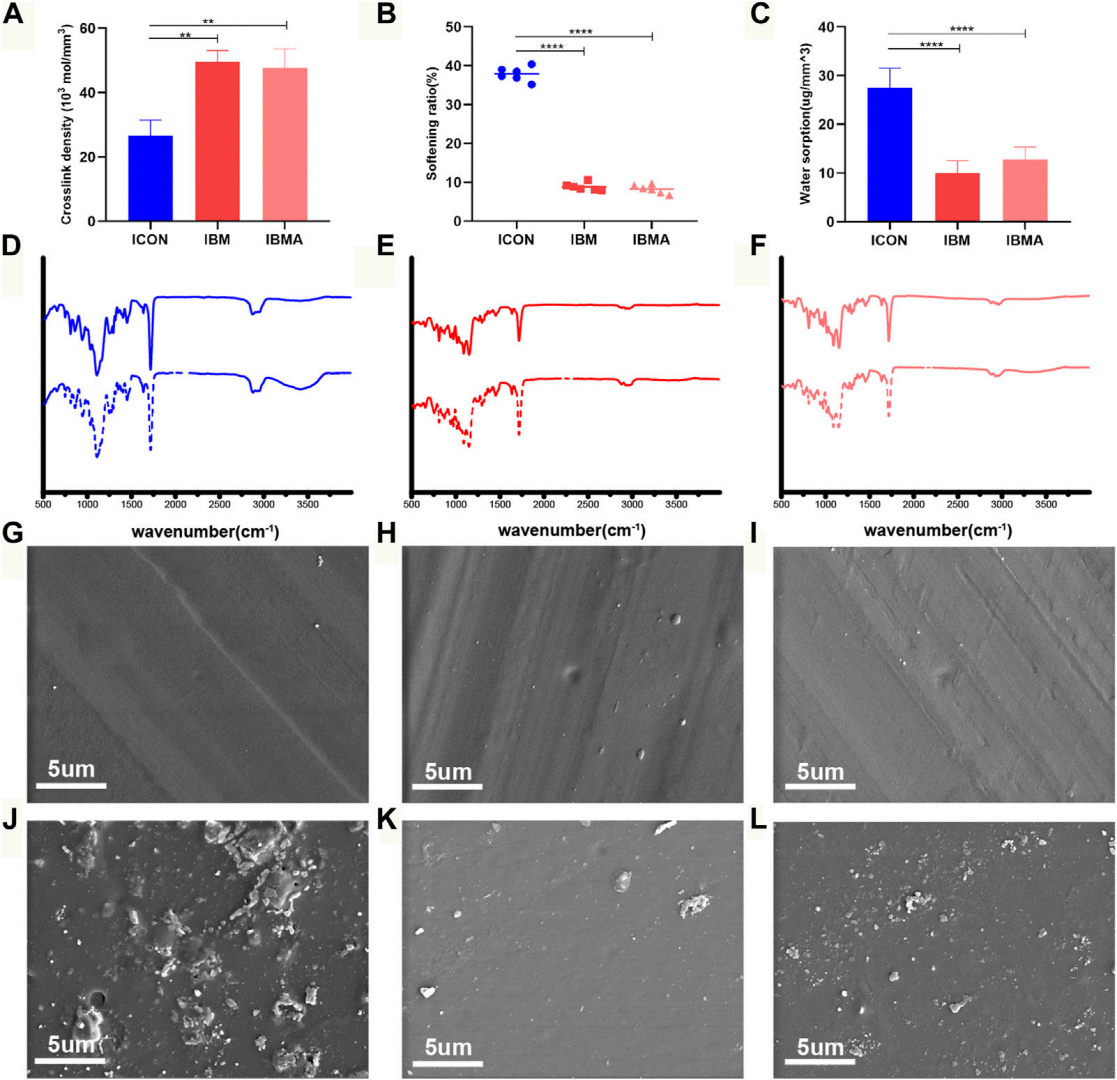
**(A)** Comparison of the crosslink density among ICON, IBM, and IBMA resins. **(B)** Comparison of the softening ratio among different resins. **(C)** Comparison of water sorption among different resins. ATR-FTIR spectra of ICON **(D)**, IBM **(E)**, and IBMA resins **(F)** before (dashed line) and after aging (dotted line). SEM image of ICON **(G)**, IBM **(H)**, and IBMA resins **(I)** before aging. SEM image of ICON **(J)**, IBM **(K)**, and IBMA resins **(L)** after aging. Error bars represent SD. **p < 0.01; ****p < 0.0001.

Measurements calculated from DMA analysis showed that the crosslink density of the IBMA resin and IBM resin was (47.77 ± 5.76) ×10^3^ mol/mm^3^ and (49.61 ± 3.48) ×10^3^ mol/mm^3^, respectively, which was higher than that in the ICON group [(26.65 ± 4.79) ×10^3^ mol/mm^3^], and the difference was statistically significant (*p* < 0.01) (Figure 2A). The softening ratio of the IBMA resin and IBM resin was 8.25% ± 1.13% and 8.82% ± 0.96%, respectively, which was lower than that in the ICON group (37.88% ± 1.82%), and the difference was statistically significant (*p* < 0.0001) (Figure 2B). Furthermore, the water sorption of the IBMA resin and IBM resin was 12.79 ± 2.56 ug/mm^3^ and 10.01 ± 2.46 ug/mm^3^, respectively, which was lower than that in the ICON group (27.49 ± 4.03 ug/mm^3^), and the difference was statistically significant (*p* < 0.0001) (Figure 2C). The ATR-FTIR spectrum showed that the peak of the hydroxyl group around 3,400 cm^−1^ on the surface of the ICON resin increased significantly after simulated aging, while the IBMA and IBM resins did not present similar changes (Figures 2D–F). Furthermore, in SEM images, the ICON surface had poor structural integrity and increased roughness after simulated aging, whereas the IBMA resin had a slightly rough surface and the IBM resin exhibited no noticeable change (Figures 2G–L).

### 3.3 Antibacterial effect of the IBMA resin under simulated aging

**FIGURE 3:**
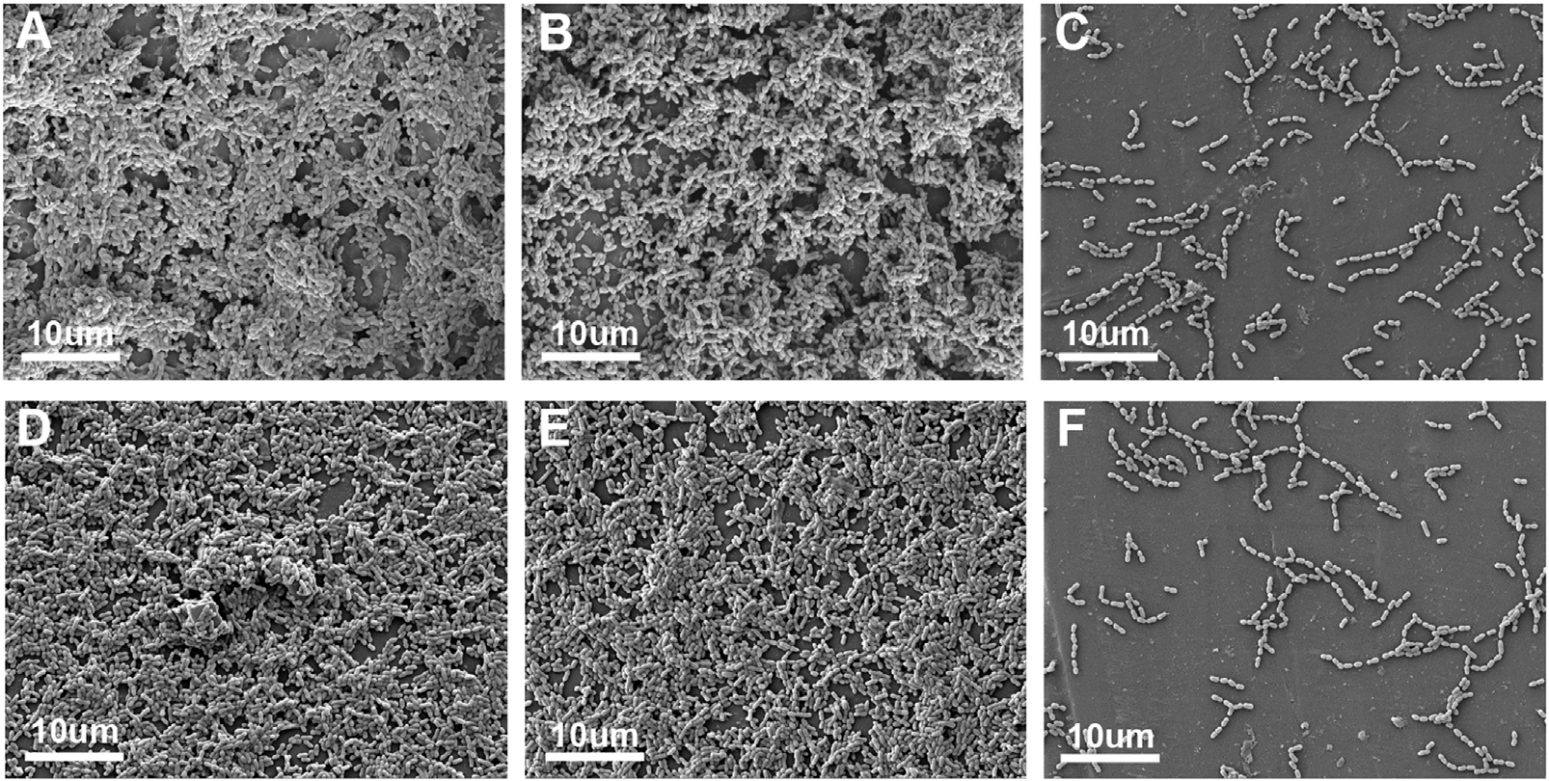
SEM image of the UA159 biofilm on the surface of ICON **(A)**, IBM **(B)**, and IBMA resins **(C)** before aging. SEM image of the UA159 biofilm on the surface of ICON **(D)**, IBM **(E)**, and IBMA resins **(F)** after aging.

UA159 biofilm adhesion on the surface of each group is shown in Figure 3. Despite simulated aging, the IBMA resin still demonstrated a reduced biofilm adhesion.

### 3.4 Infiltration performance and recovery effect of the IBMA resin on the demineralized enamel

**FIGURE 4:**
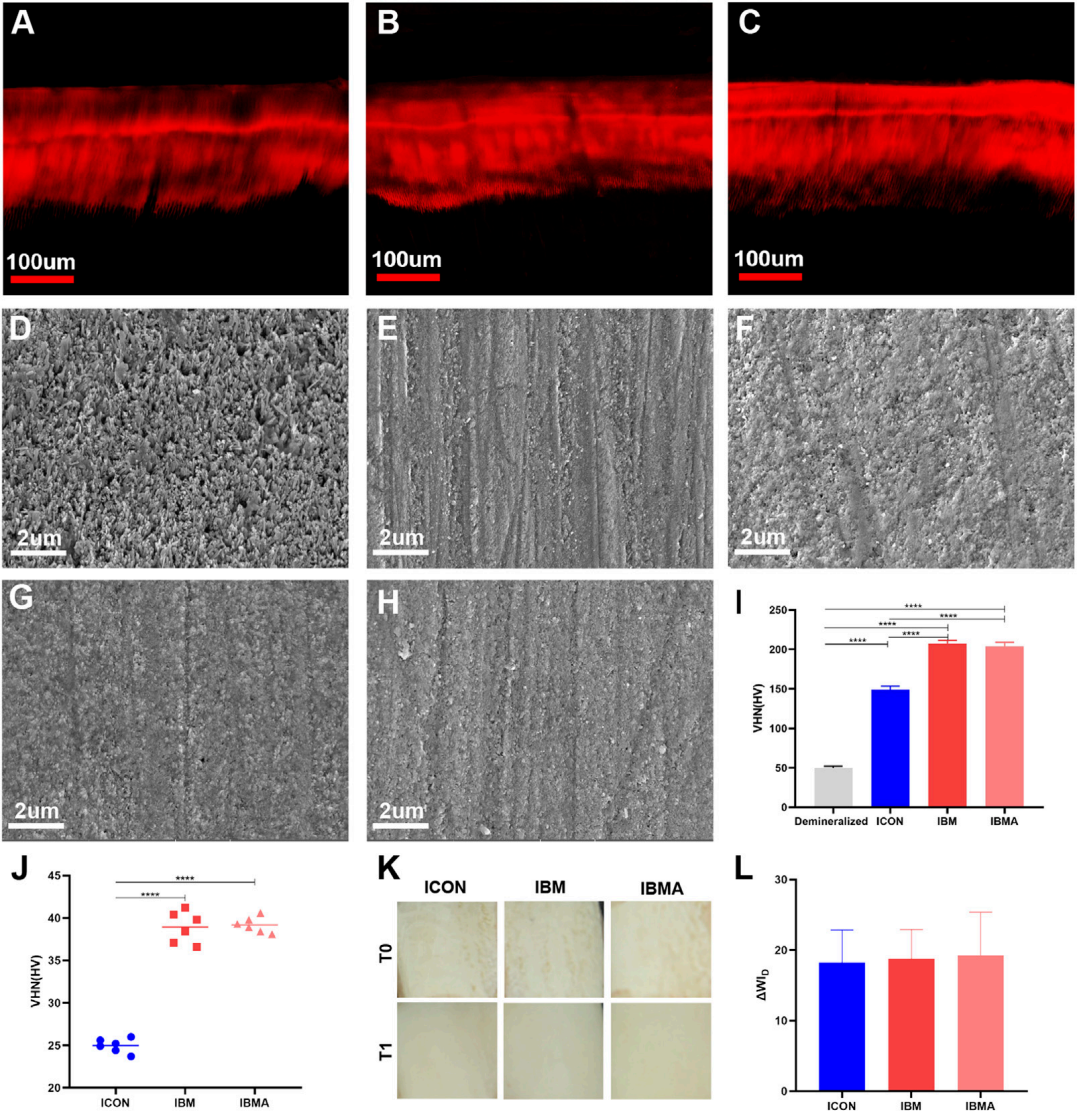
CLSM image of the infiltration depth of ICON **(A)**, IBM **(B)**, and IBMA resins **(C)** on the demineralized enamel. SEM image of the untreated demineralized enamel surface **(D)**, sound enamel surface **(E)**, ICON-infiltrated **(F)**, IBM-infiltrated **(G)**, and IBMA-infiltrated **(H)** enamel surface. **(I)** Vickers microhardness of the untreated demineralized enamel and different infiltrated lesions. **(J)** Comparison of Vickers microhardness among different resins. **(K)** Images of the demineralized enamel of different groups before (T0) and after resin infiltration (T1). **(L)** Comparisons of ΔWI_D_ among different groups after resin infiltration. Error bars represent SD. ****p < 0.0001.

The infiltration depth of the IBMA or IBM resin was not statistically different from that of the ICON resin (Figures 4A–C, Supplementary Tables S3, S4). Moreover, the SEM images showed that the IBMA resin, IBM resin, and ICON occluded the exposed micropores, restoring surface morphology close to the sound enamel (Figures 4D–H). The microhardness of the demineralized enamel was 49.93 ± 2.21 HV, but after being treated with ICON, IBM resin, or IBMA resin, the value increased to 149.0 ± 4.54 HV, 207.3 ± 4.43 HV, and 204.0 ± 5.12 HV, respectively. The difference among other groups was statistically significant (*p* < 0.0001), except between the IBM and IBMA groups (Figure 4I). The microhardness of IBMA and IBM resins was 39.18 ± 0.92 HV and 38.92 ± 1.85 HV, respectively, which was higher than that of ICON (24.97 ± 0.83 HV), and the difference was statistically significant (*p* < 0.0001) (Figure 4J). After being treated with the IBMA or IBM resin, ΔWI_D_ of the chalky demineralized area was 19.23 ± 6.11 and 18.80 ± 4.09, respectively, which was significantly more than the WAT threshold (2.62 WI_D_ units), demonstrating excellent esthetic improvement, similar to ICON (ΔWI_D_ = 18.25 ± 4.59) (Figures 4K,L).

### 3.5 Surface morphology and color stability of IBMA-infiltrated areas after simulated aging

**FIGURE 5:**
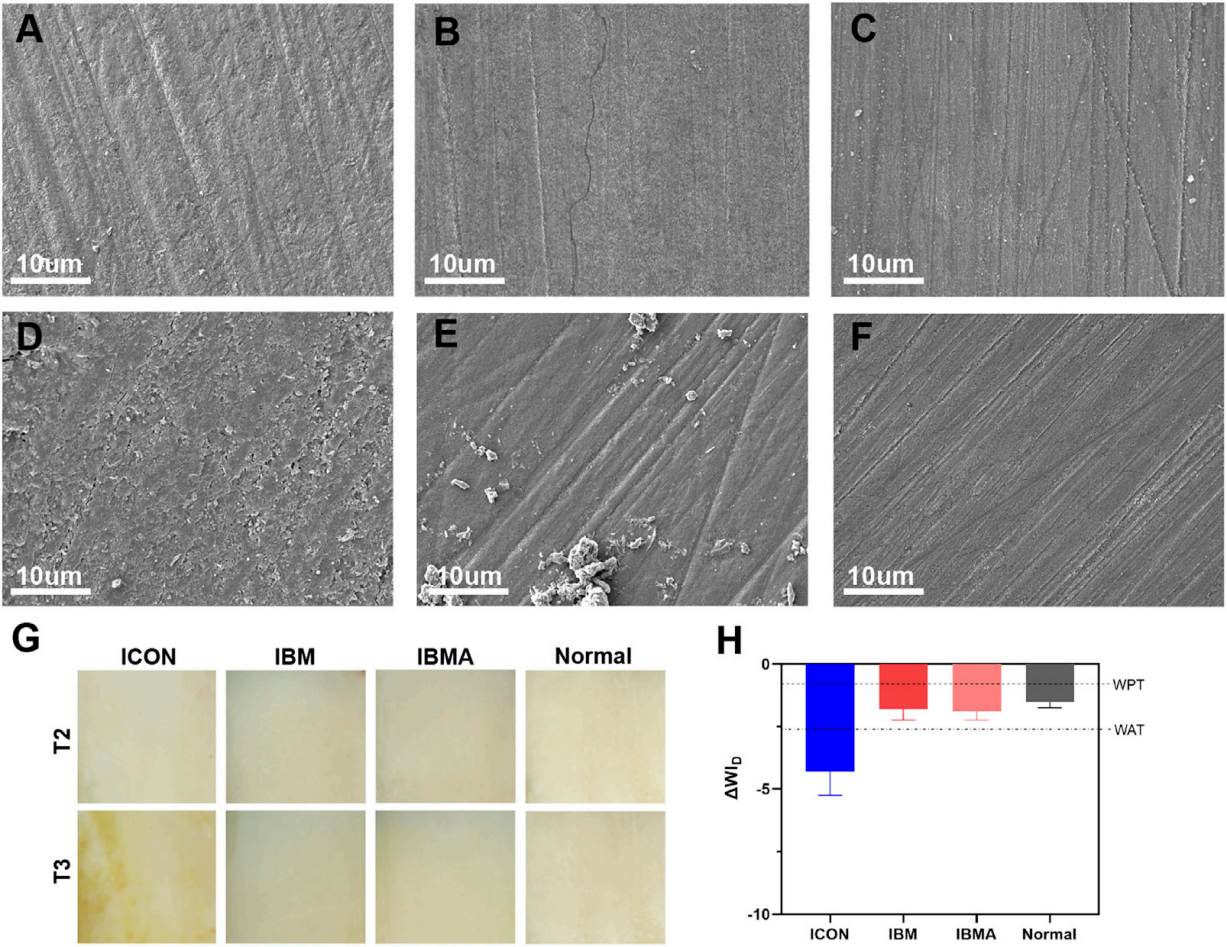
SEM image of ICON-infiltrated **(A)**, IBM-infiltrated **(B)**, and IBMA-infiltrated **(C)** enamel surfaces before aging. SEM image of ICON-infiltrated **(D)**, IBM-infiltrated **(E)**, and IBMA-infiltrated **(F)** enamel surfaces after aging. **(G)** Images of the infiltrated enamel of different groups before (T2) and after the staining challenge (T3). **(H)** Comparisons of ΔWI_D_ among different groups after the staining challenge. WPT: whiteness 50%:50% perceptibility (0.72 WI_D_ units); WAT: whiteness 50%:50% acceptability (2.62 WI_D_ units). Error bars represent SD.

According to SEM images, microcracks appeared on the surface of the ICON-treated area after simulated aging, and there was no obvious change on the surface of the IBMA and IBM groups (Figures 5A–F). After staining, ΔWI_D_ of the IBMA-treated or IBM-treated area was −1.90 ± 0.34 and −1.81 ± 0.42, respectively, which was similar to the normal enamel (−1.52 ± 0.23), between WPT and WAT, reflecting good color stability. However, ΔWI_D_ of the ICON-treated area was −4.30 ± 0.95, higher than the WAT threshold, which indicated obvious color alteration (Figures 5G,H).

## 4 Discussion

### 4.1 Preparation and preference of IBM-based antibacterial infiltration resins

Although the clinical application of the available infiltration resin conforms to the development concept of minimally invasive dentistry, the TEGDMA-based (78%) material has problems, such as poor biosafety and ease of degradation, due to its own characteristics (Gölz et al., 2016; Chen et al., 2019). This study aimed to fundamentally solve TEGDMA’s inherent defects and impart the ability to inhibit biofilm adhesion by fabricating a TEGDMA-free infiltrant based on the IBM monomer and MPC agent. First, the IBM monomer was successfully synthesized using isosorbide and methacryloyl (Figure 1A). Next, the rheological examination showed that its viscosity was only slightly higher than that of TEGDMA (Figure 1B), which is consistent with the results of previous studies (Herrera-González et al., 2019). The monomer’s low viscosity is the premise for fabricating the infiltration resin. The low-viscous resin could penetrate microporous lesions, thereby partially restoring the enamel structure, curbing the progression of caries, and improving the enamel color (Paris et al., 2007b; Paris and Meyer-Lueckel, 2010).

To achieve a preferential selection of materials, the comparison regarding antibiofilm adhesion was conducted between IBM-based infiltration resins and the commercial product, ICON. The protein adsorption test (Figure 1C), crystal violet assay (Supplementary Figure S2), and surface SEM analysis (Figures 1D–H) of the UA159 biofilm indicated that IBM-based resins containing 2% MPC and 3% MPC both exhibited obvious biofilm-repellent properties. The reason can be attributed to the role of MPC, whose anionic and cationic groups form a free-water layer to resist nonspecific protein adsorption (Cheng et al., 2017). Next, considering that the infiltration resin is mostly applied to children and adolescents, its biosafety needs closer attention. Using CCK-8 assay, the IBM-based resins showed significantly greater cell viability than ICON (Figure 1I), without any dilution of the conditioned medium. Specifically, IBM groups containing 0%, 1%, and 2% MPC showed more than 70% cell viability, meeting the non-cytotoxicity requirements based on the ISO standard (ISO10993-5, 2009). This prominent advantage could be explained as twofold, that is, the compatibility of the IBM-based polymer is good, and the IBM monomer with a rigid structure is not easy to be released from the material. In contrast, long-chain and flexible TEGDMA could be easily freed from the matrix of ICON, disrupt cell homeostasis, and trigger a significant cytotoxic response (Geurtsen and Leyhausen, 2001; Bakopoulou et al., 2011). According to Soares et al., the ICON resin exhibited modest cytotoxicity to human dental pulp stem cells (Gölz et al., 2016), even though the conditioned medium was prepared using a 1 ug/ml ratio, far below our 0.2 g/ml ratio. Therefore, despite adding a certain concentration of antibacterial agents, the aforementioned results suggest that novel IBM-based resins can overcome TEGDMA’s poor biosafety. Based on comprehensive antibacterial performance and cytocompatibility considerations, the IBM-based resin containing 2% MPC was determined to be the preferred antibacterial infiltration resin, hereinafter referred to as IBMA. Afterward, a series of comparative studies were conducted with the commercial ICON resin to evaluate IBMA’s potential and benefits of clinical application.

### 4.2 Hydrolysis-resistant performance of the IBMA resin under simulated aging

In the saliva environment, ester bonds in the existing infiltration resin are prone to be attacked and hydrolyzed, causing degradation of the polymer network. As a result, it would compromise the blocking effect on demineralized lesions, which would affect the color stability of infiltrated areas and weaken the control of early decay, resulting in the recurrence of caries (Zhao and Ren, 2016; Chen et al., 2019). Therefore, hydrolysis resistance is crucial for the infiltration resin to improve its long-term clinical performance and avoid failure of minimally invasive treatments. As previously described, we imagined the IBM-derived resin to demonstrate hydrolysis resistance based on the rational assumption of the polymer’s crosslink degree. As expected, both the direct measurement of DMA analysis and the indirect softening ratio results confirmed that the crosslink density of the IBMA polymer network was superior to that of ICON (Figures 2A,B). As a result of its short-chain and rigid dual five-numbered ring structure, the IBM monomer does not cause local defects such as cyclization and entanglement during polymerization, unlike long-chain TEGDMA (Park et al., 2011; Barszczewska-Rybarek, 2019). The higher the crosslink density, the higher is the number of effective crosslink points per unit volume of the polymer, indicating that the material has good structural stability. Given that the TEGDMA-based infiltration resin was susceptible to absorbing water, inducing an ester bond breakage (Zhao and Ren, 2016; Chen et al., 2019), we also further evaluated the water sorption of the IBMA resin and found that it was dramatically smaller than that in the ICON resin (Figure 2C). The phenomenon may be explained as follows: TEGDMA, which contains triethylene glycol units, has a stronger hydrophilicity than the IBM monomer. Furthermore, IBMA’s denser polymer network is less susceptible to water penetration (Park et al., 2011; Łukaszczyk et al., 2013). Low water uptake could reduce the risk of polymer’s ester bonds being vulnerable to breaking since it undergoes less internal stress caused by water sorption than that of the TEGDMA-based resin (Zhao and Ren, 2016).

In order to reflect the antihydrolysis performance of the material after long-time oral service as much as possible, we simulated the saliva environment and accompanying temperature fluctuation through thermocycling. The results of the Fourier transform infrared spectroscopy demonstrated that the hydroxyl group of the IBMA resin at around 3,400cm^−1^ had no significant change after aging (Figure 2F), indicating that no hydrolytic reaction occurred, while the ICON resin had an obvious peak of the hydroxyl group (Figure 2D), indicating that its ester bond was attacked and hydrolytically cleaved to generate degradation products such as TEG, in accordance with the previous research (Chen et al., 2019). Consistently, the microhardness of IBMA did not decrease significantly after aging (Supplementary Figure S4), and its surface microstructure was only slightly rough, close to the initial state (Figures 2I,L). However, the microhardness of ICON decreased significantly (Supplementary Figure S4), and its surface integrity was poor with increased roughness (Figures 2G,J). These comparisons highlighted that IBMA has better hydrolysis resistance than ICON. Thus, the novel infiltration resin successfully addressed the TEGDMA system’s inherent problem of easy degradation. Consequently, micropore sealing can be maintained in future applications, thereby improving caries control and color stability.

### 4.3 Antibacterial effect of the IBMA resin under simulated aging

In addition to hydrolysis resistance, infiltration resin optimization also focuses on how to reduce the adverse effect of plaque accumulation on the treated area’s stability. Since the available infiltration resin lacks antibacterial function, the resin–hydroxyapatite complex would be disrupted when it experiences plaque attack (Kielbassa et al., 2020), which no longer effectively inhibits the diffusion of cariogenic factors. Thus, it is necessary to impart durable antibacterial property to the infiltrant. The aforementioned findings have preliminarily confirmed that IBMA could repel the adhesion of the UA159 biofilm. Therefore, we further evaluated whether the IBMA resin has lasting antibacterial activity after artificial aging. It turned out that IBMA’s ability to resist biofilm adhesion remained the same, regardless of before or after thermocycling (Figure 3). The durable biofilm-repellent effect could be attributed to MPC’s methacrylate groups covalently bonding to the resin matrix during photocuring polymerization, as well as IBMA’s structural stability against hydrolysis. Zhang et al. (2018) also reported a long-lasting antibacterial effect when adding MPC to the adhesive. This characteristic could reduce the risk of redemineralization of infiltrated areas and accelerate the degradation of the polymer network caused by bacterial acid production and microbial-releasing esterases, finally contributing to infiltration resin’s long-term stability. Considering the biosafety, hydrolysis resistance, and antibacterial properties of the IBMA resin, we would further explore its infiltration performance and effectiveness on the demineralized enamel.

### 4.4 Infiltration performance and recovery effect of the IBMA resin on the demineralized enamel

Whether infiltration resin can penetrate and fill the micropores is the prerequisite for restoring demineralized enamel. Paris found that resins with low viscosity and low contact angle are more likely to infiltrate into the enamel micropores (Paris et al., 2007b; Paris and Meyer-Lueckel, 2010). Thus, we first analyzed the rheology and wettability of the IBMA resin. Its viscosity and contact angle on the bovine enamel (Supplementary Figures S5, S6; Supplementary Tables S1, S2) preliminarily suggested that IBMA has the ability to enter into the lesion by capillary force. Afterward, we further evaluated its actual infiltration depth in enamel lesions, which is often used to represent the range of the infiltration resin encapsulating weak demineralized crystals, closely related to the effect of infiltration treatment. With the indirect staining method modified by Paris (Paris et al., 2009), we found that the infiltration depth of the IBMA resin was not statistically different from that of ICON (Figures 4A–C, Supplementary Tables S3, S4), which indicated that IBMA could penetrate micropores and form a similar resin-porous enamel complex. The barrier can prevent cariogenic factors from extending deep, restore the enamel structure partially, and regain enamel translucency. Although its viscosity and contact angle were slightly higher than those in ICON, the IBMA resin still exhibited a similar infiltration ability. The reason may be that 15% HCl gel can fully open the micropores of the enamel surface after 2 min of acid etching (Meyer-Lueckel et al., 2007), favorable for penetration, and 5 min of application is enough for it to reach the demineralized lesions through capillary force (Meyer-Lueckel et al., 2011).

Furthermore, we analyzed IBMA’s effect on the enamel surface integrity, strength, and translucent appearance in the demineralized area. Benefiting from the formation of the resin-porous enamel complex, the IBMA resin achieved the restoration of enamel surface morphology and strengthening of the lesion structure (Figures 4D–H), which can prevent enamel disintegration and further demineralization in deep regions. We noticed that the IBMA resin brought about a higher enamel microhardness than ICON (Figure 4I), and the reason lies in the microhardness of the IBMA resin itself being higher than ICON (Figure 4J), given IBMA polymer’s high crosslink degree and IBM monomer’s rigid skeleton. According to these findings, the novel infiltration resin is more resistant to mastication pressure in the oral environment. Additionally, after being treated with the IBMA resin, the chalky demineralized area displayed an excellent instance of esthetic improvement (Figures 4K,L). IBMA’s effective penetration depth and its refractive index close to that of normal enamel (RI = 1.491, Supplementary Tables S5, S6) both led to a decrease in local light scattering, thereby regaining enamel translucency (Paris et al., 2013). In brief, IBMA achieves enamel topography and color recovery similar to the commercial product, and noteworthily, it has better performance in enhancing lesion strength, which could facilitate the infiltrated enamel enduring complex stress in the oral cavity.

### 4.5 Surface morphology and color stability of IBMA-infiltrated areas after simulated aging

One original intention of developing the novel infiltration resin is to enhance the stability of the resin-porous enamel complex under an oral environment. Since the IBMA resin has shown improved hydrolysis resistance, we speculated that its sealing effect of demineralized micropores would withstand hydrolytic degradation well. Interestingly, following the aging challenge, the surface of IBMA-infiltrated enamel lesions retained a similar morphology as that of a normal enamel (Figures 5C,F), whereas ICON-treated areas developed microcracks (Figures 5A,D). This discrepancy may stem from their respective hydrolysis-resistant performance. The IBMA resin and its bonding with enamel crystals have withstood hydrolysis challenges during simulated aging, eventually maintaining the surface integrity of treated areas. The results indicated that the IBMA resin-porous enamel crystal complex could still function as a good barrier against the entry of cariogenic factors even after aging. In other words, the IBMA resin effectively overcomes the problems of blocked-micropore exposure caused by the easy degradation of available materials (Zhao and Ren, 2016).

Many researchers believed that the existing infiltration resin is susceptible to discoloration in daily diet; hence, its long-term esthetic performance on chalky lesions is questionable (Altarabulsi et al., 2014; Zhao and Ren, 2016; Chen et al., 2019). Thus, we further explored the color stability of the treated enamel *via* staining bovine specimens with black-tea solution after simulated aging. It turned out that the IBMA group reflected good color stability, while the ICON-treated area showed a significant decrease in WI_D_ (Figures 5G,H). It could be explained as a discrepancy in the surface integrity and polymer’s water sorption between two materials (Park et al., 2011; Zhao and Ren, 2016). First, the IBMA-treated areas did not have microcracks or microfissures after aging. Thus, pigment adsorption was minimal. Second, compared to ICON, IBMA’s water sorption is lower, resulting in less pigment entering IBMA’s network along with water uptake. Generally, the specimens of the IBMA group showed favorable color stability after the staining challenge. According to the present study, the novel infiltration resin can reduce the risk of minimally invasive dental treatment failure due to material degradation and maintain esthetic improvement over time.

**FIGURE 6:**
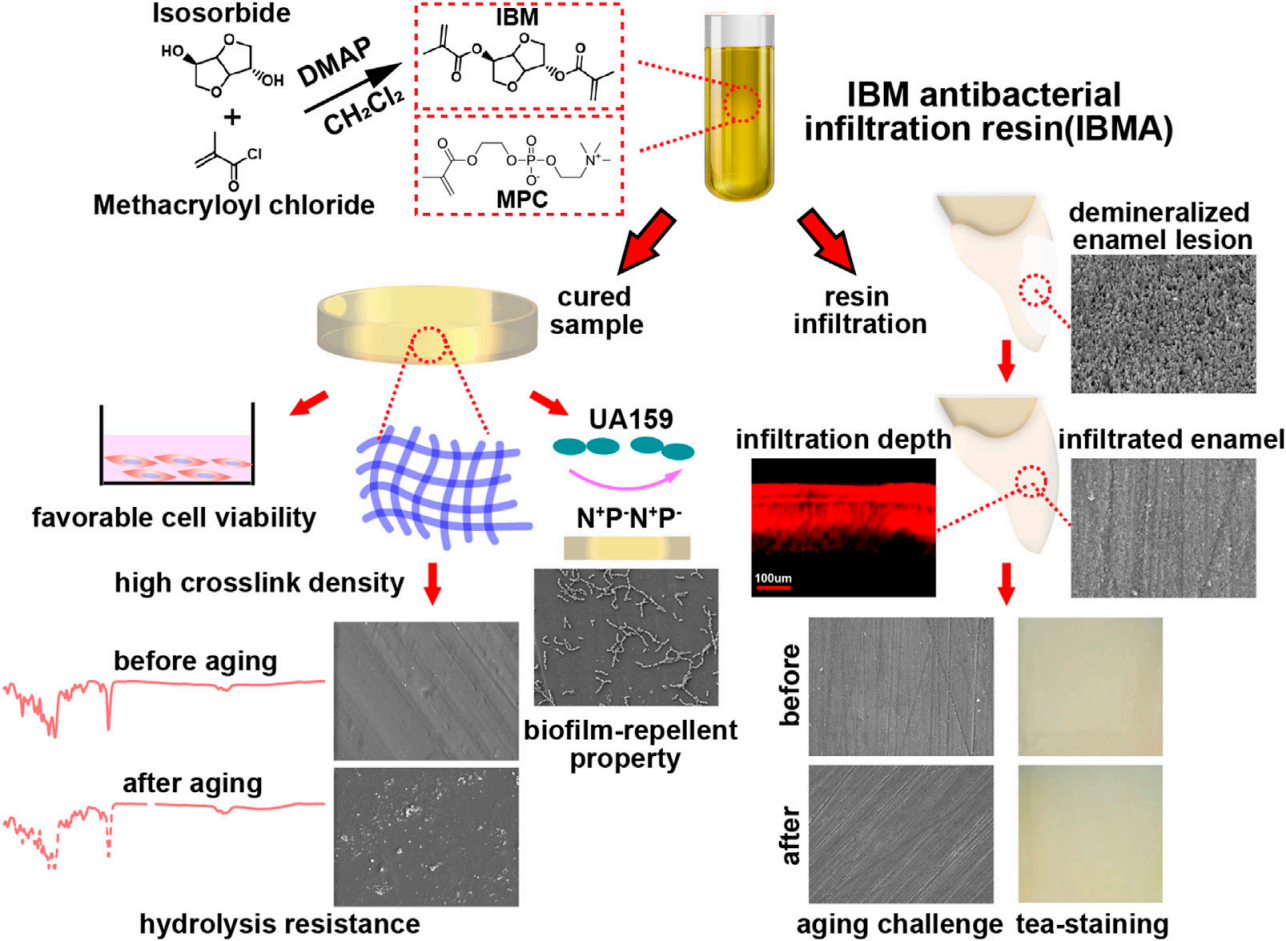
Schematic illustration of the preparation and characteristics of the novel IBMA resin.

The bioderived IBM monomer serves as novel infiltration resin’s main component, endowing the material with favorable cell viability. IBMA can exert a durable antibiofilm adhesion effect through MPC’s protein-repellent property and its bonding to the resin matrix during light-curing polymerization. Benefiting from low viscosity and good wettability, the IBMA resin can effectively penetrate demineralized lesions and form a resin-porous enamel complex, thereby restoring the morphology, strength, and color of the damaged enamel. Finally, the novel infiltration resin demonstrates excellent hydrolysis resistance because of the polymer network’s characteristics of high crosslink density and low water sorption. Consequently, its treated areas have demonstrated surface integrity and color stability even after aging (Figure 6).

## 5 Conclusion

To sum up, IBMA as a novel infiltration resin of the non-TEGDMA system has been successfully developed, containing a biobased IBM monomer as the main component followed by 2% MPC. The IBMA resin has infiltration properties similar to commercial products and could achieve the recovery effect of the demineralized enamel. In addition, the material exhibits excellent cytocompatibility, favorable hydrolysis resistance, and durable biofilm-repellent effect. Given these characteristics, the IBMA resin is expected to avoid biological risks of the existing material, improve the stability of early caries hampering or esthetic improvement in treated lesions, and reduce the occurrences of local redemineralization caused by plaque attachment. The advantages imply that the novel infiltration resin has potential application prospects and important clinical significance.

## Data Availability

The raw data supporting the conclusion of this article will be made available by the authors, without undue reservation.
