# Supplementary material for: A novel dental infiltration resin based on isosorbide-derived dimethacrylate with high biocompatibility, hydrolysis resistance, and antibacterial effect
Source: Front Bioeng Biotechnol. 2022 Nov 10;10:1049894. doi: 10.3389/fbioe.2022.1049894 (PMC9685411; doi:10.3389/fbioe.2022.1049894)
Supplement: Supplementary file 1 [file Table1.DOCX]

**A novel dental infiltration resin based on isosorbide-derived dimethacrylate with high biocompatibility, hydrolysis resistance and antibacterial effect**

**Appendix**

**2.Materials and Methods**

*2.1 Preparation and preference of IBM-based antibacterial infiltration resins*

**Micro bicinchoninic acid (BCA) method**

Cured specimens (N=6) from each group were immersed in phosphate-buffered saline (PBS, Biosharp) for 2 h, and then soaked in 4.5 g/l bovine serum albumin solution (BSA, Sigma-Aldrich) for 6 h at 37 °C. After rinsing with PBS by stirring at a speed of 300 r/min for 5 min, the discs were immersed in PBS solution containing 1% sodium dodecyl sulfate (SDS, Solaibao), and sonicated for 30 min at room temperature to detach the BSA adsorbed onto the surface completely. A protein analysis kit (BCA protein assay kit; Fisher Scientific) was used to determine the BSA concentration in the SDS solution. Briefly, 25 ul of the SDS solution from each group and 200 ul of the BCA working reagent were mixed in a 96-well plate and incubated at 37°C for 1h. Then, absorbance at 562nm was detected by a microplate reader (Multiskan FC, Thermo Fisher). Protein concentration was quantified according to the standard curve of BCA-protein concentration, by which the amount of protein adsorbed on the disc surface was calculated.

**Crystal violet assay**

Six specimens from each group were fixed with 1 ml methyl alcohol for 15 min, and transferred to a new 12-well plate after rinsed with PBS. Then they were immersed in 1 ml 0.1 % crystal violet solution (C8470; Solarbio) for 5 min. After rinsing with PBS to remove residual dye, the discs were transferred to another 12-well plate. Then, 2ml 95 % ethanol solution was added to each well, and the plate was shaken horizontally for 45 min at room temperature. 100ul shaken ethanol solution from each well was diluted with 95 % ethanol solution to 200 μl and transferred to a 96-well plate. Absorbance of the solution at 595 nm were measured under a microplate reader (Multiskan FC, Thermo Fisher).

*2.2 Hydrolysis-resistant performance of IBMA resin under simulated aging*

**Water sorption** **tests of different resins**

Disc-shaped cured samples of ICON, IBM and IBMA resin (N=6) were prepared and placed in a desiccator and repeatedly weighed until constant mass was obtained. Then they were immersed in 10ml deionized water individually at 37 °C. After 7 days of water storage, the samples were dried with absorbent paper and weighed on an analytical scale to obtain (m_1_). Afterwards, they were placed in the desiccator and dried until the final constant mass was obtained (m_2_). The sorption (So) values were calculated using the following formula: So = m_1_-m_2_/v, and v is the volume of the specimen.

**Vickers microhardness of different resins before and after thermocycling**

Vickers microhardness of cured specimens (N=6) from each group were measured on four quadrants of the surface using a Vickers indenter (HXD-1000TMC/LCD, Taiming), under a load of 50 g for 10 s, obtaining initial VHN (VHN_initial_). Thereafter, the samples were subjected to thermocycling and the microhardness values after aging were recorded as VHN_aging_. The extent of microhardness alteration of each group- ΔVHN (%) =[(VHN_initial_-VHN_aging_)/VHN_initial_] *100%.

*2.4 Infiltration performance and recovery effect of IBMA resin on demineralized enamel*

**Preparation of bovine specimens**

Enamel-dentin specimens (6 mm*6mm, 4 mm thick) were obtained from the labial surfaces of sound bovine central incisors by a low-speed saw (Isomet, Buehler) under water-cooling. Teeth with surface defects and discoloration were excluded. All specimens were further polished by 400-grit, 800-grit and 1200-grit SiC papers with water irrigation to obtain a flat enamel and dentine surface. Then they were ultrasonic cleaned for 5 min to remove smear layer. The prepared specimens were stored in distilled water under refrigeration at 4 °C until required.

**Viscosity and contact angle of different resins**

Viscosity of different resins was determined using the rotary rheometer (MCR302, Anton paar). Contact angle measurements were determined using a goniometer with a drop shape analysis software (DSA 10, Krüss). Droplets of each material were placed on the flattened bovine enamel surface by means of a micro syringe. After 10 s an image was recorded and analyzed using the accompanying software.

**Preparation of artificial demineralized bovine specimens**

Selection of bovine specimens was in terms of baseline surface microhardness, minimizing the variation between them (10% VHN). Microhardness was performed using a microhardness tester (HXD-1000TMC/LCD, Taiming) with a load of 200 g for 15 s. Subsequently, acid resistant nail varnish was applied to cover surface of each specimen, leaving unprotected labial enamel areas. Demineralized enamel lesions were induced by immersing each specimen into the demineralizing solution for three weeks at 37℃, changed every two days. The demineralizing solution used was composed of 0.01mmol/l NaF ,2.2 mM calcium chloride (CaCl_2_), 2.2 mM potassium phosphate (KH_2_PO_4_) and 50 mM acetic acid with pH equal to 4.5(Meligy et al., 2020; Yu et al., 2020). Each specimen was inspected visually to ensure that demineralized lesions were successfully created on the enamel surfaces, identified as an opaque and chalky white area.

**Confocal laser scanning microscopy (CLSM) evaluation of infiltration depth on demineralized enamel**

Artificial demineralized bovine specimens were prepared and randomly distributed among the ICON, IBM and IBMA group. Chalky enamel lesions from each group were etched for 2 min using 15% hydrochloric acid gel. After drying, specimens were stained with ethanolic tetramethylrhodamine isothiocyanate 0.1% (TRITC; Sigma Aldrich) for 12 h. Subsequently, specimens were dried using compressed air for 10 s, wetted with absolute ethanol for 30 s and dried again for 10 s. Then each infiltrant was applied onto the lesion surface for 5 min and light cured for 40 s. Specimens were sectioned perpendicularly to the lesion surfaces in order to obtain slices of 1mm in thickness. Unbound rhodamine dye was bleached by immersion slices in hydrogen peroxide (30%) for 12 h at 37 ° C. Subsequently, they were washed with distilled water for 60 s.

**Color evaluation and measurement of refractive index**

The contact-type dental spectrophotometer has a 5.0mm-diameter aperture size. Values (L*, a*, b*) were measured by a trained operator who was blinded with respect to treatment group in darkness. For every measurement, the device was conducted an automatic white balance on its calibration block. Measurements were done in triplicate and average values of color coordinates were recorded. The L* value represents the lightness of the color (0 = black, 100 = white), the a* value represents the color between greenness (negative a*) and redness (positive a*), and the b* value represents the color between blueness (negative b*) and yellowness (negative b*). Refractive index of liquid samples (N=3) from ICON, IBM, IBMA group was calculated using WYV refractometry.

1. **Results**


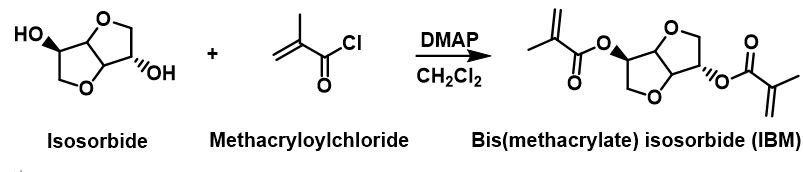


**Figure S1. Chemical reaction process of IBM monomer synthesis**


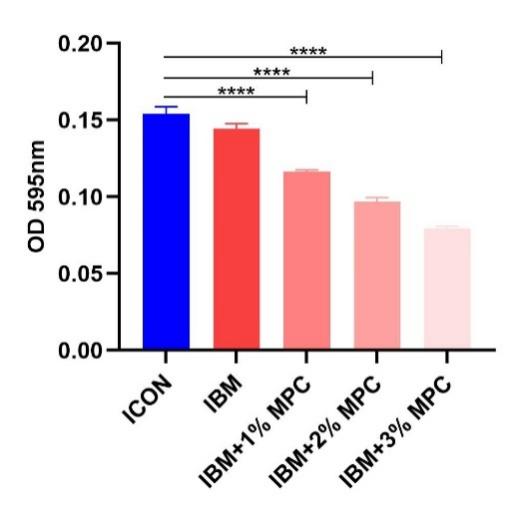


**Figure S2. Comparison of UA159 biofilm amount on the surface of different resins via crystal violet assay.** The amount of biofilm adhesion on the surface of IBM-based resins containing MPC was significantly lower than that of ICON, and the difference was statistically significant (*P*<0.0001).

**Figure S5. Viscosity of ICON, IBM and IBMA resin**


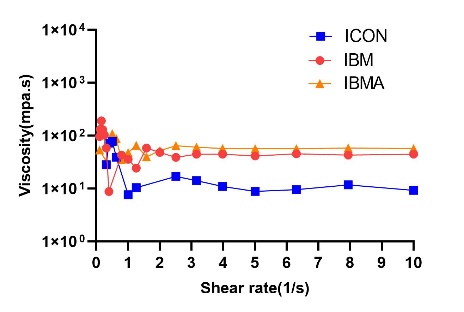

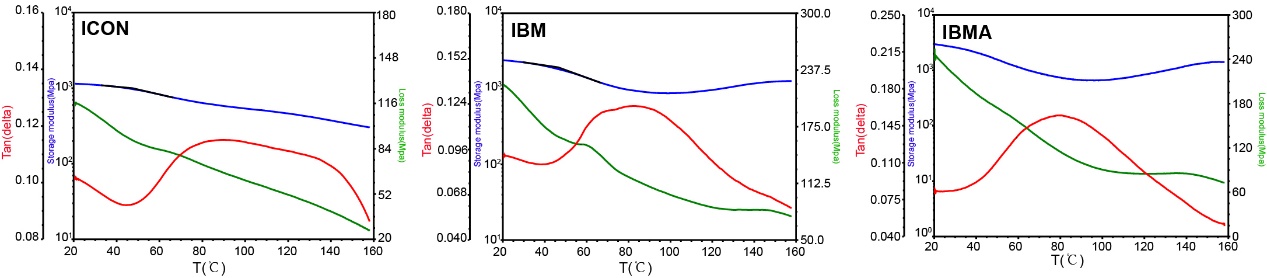


**Figure S3. Tangent curve, storage modulus curve (E’) and loss modulus curve (E’’)**

**of different resins by DMA analysis**


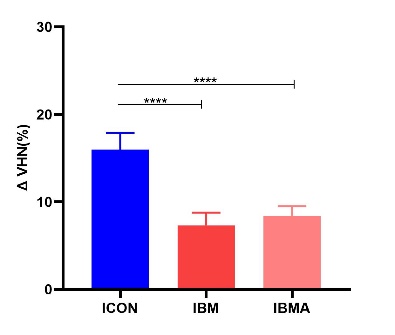


**Figure S4. Comparison of the extent of microhardness alteration of different resins after aging.** Reduced ratio of surface microhardness of IBMA resin and IBM resin after aging are 8.4±1.12%, 7.32±1.46%, respectively, smaller than ICON group (15.96±1.96%), and the difference was statistically significant (*P*<0.0001).


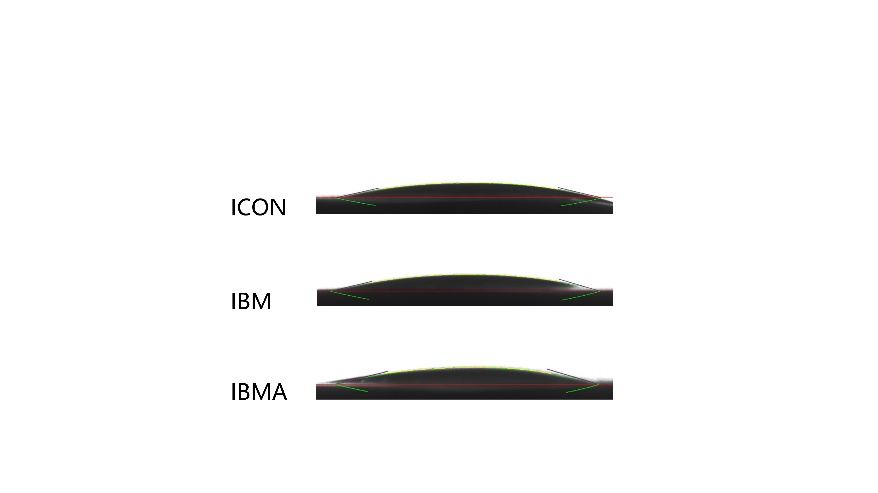


**Figure S6. Contact angle of ICON, IBM and IBMA resin on bovine enamel**

| **Table S1. Descriptives of contact angle (°) of different resins** | | | | | | | |
| --- | --- | --- | --- | --- | --- | --- | --- |
| GROUP | | Mean | | SD | |  | |
| ICON |  | 11.567 |  | 0.551 |  |  |  |
| IBM |  | 12.430 |  | 0.446 |  |  |  |
| IBMA |  | 12.830 |  | 0.316 |  |  |  |

| **Table S2. Comparison of contact angle among different resins** | | | | | | | | | | |
| --- | --- | --- | --- | --- | --- | --- | --- | --- | --- | --- |
|  | |  | | Mean Difference | | SE | | t | | p |
| ICON |  | IBM |  | -0.863 |  | 0.366 |  | -2.361 |  | 0.122 |
|  |  | IBMA |  | -1.263 |  | 0.366 |  | -3.455 |  | 0.031^*^ |
| IBM |  | IBMA |  | -0.400 |  | 0.366 |  | -1.094 |  | 0.552 |

*: p<0.05

| **Table S3. Descriptives of infiltration depth(um) of different resins on demineralized enamel** | | | | | | | |
| --- | --- | --- | --- | --- | --- | --- | --- |
| GROUP | | Mean | | SD | |  | |
| ICON |  | 213.580 |  | 23.009 |  |  |  |
| IBM |  | 208.300 |  | 27.681 |  |  |  |
| IBMA |  | 203.920 |  | 22.544 |  |  |  |

| **Table S4. Comparison of infiltration depth(um) among different resins** | | | | | | | | | | | |
| --- | --- | --- | --- | --- | --- | --- | --- | --- | --- | --- | --- |
|  | |  | | Mean Difference | | SE | | t | | p | |
| ICON |  | IBM |  | 5.280 |  | 6.331 |  | 0.834 |  | 0.683 |  |
|  |  | IBMA |  | 9.659 |  | 6.331 |  | 1.526 |  | 0.284 |  |
| IBM |  | IBMA |  | 4.379 |  | 6.331 |  | 0.692 |  | 0.769 |  |

| **Table S5. Descriptives of refractive index of different resins** | | | | | | | |
| --- | --- | --- | --- | --- | --- | --- | --- |
| GROUP | | Mean | | SD | |  | |
| ICON |  | 1.467 |  | 1.528e -4 |  |  |  |
| IBM |  | 1.491 |  | 5.774e -5 |  |  |  |
| IBMA |  | 1.491 |  | 1.000e -4 |  |  |  |

| **Table S6. Comparison of refractive index among different resins** | | | | | | | | | | | |
| --- | --- | --- | --- | --- | --- | --- | --- | --- | --- | --- | --- |
|  | |  | | Mean Difference | | SE | | t | | p | |
| ICON |  | IBM |  | -0.024 |  | 9.027e -5 |  | -266.247 |  | <0.0001^****^ |  |
|  |  | IBMA |  | -0.024 |  | 9.027e -5 |  | -266.616 |  | <0.0001^****^ |  |
| IBM |  | IBMA |  | -3.333e -5 |  | 9.027e -5 |  | -0.369 |  | 0.928 |  |

**References**

Meligy, O., Alamoudi, N.M., Ibrahim, S., Felemban, O.M., and Al-Tuwirqi, A.A. (2020). Effect of resin infiltration application on early proximal caries lesions in vitro. *Journal of dental sciences*.

Yu, J., Huang, X., Zhou, X., Han, Q., and Cheng, L. (2020). Anti-caries effect of resin infiltrant modified by quaternary ammonium monomers. *Journal of Dentistry* 97**,** 103355.
